# A National Study on Nurses’ Exposure to Occupational Violence in Lebanon: Prevalence, Consequences and Associated Factors

**DOI:** 10.1371/journal.pone.0137105

**Published:** 2015-09-10

**Authors:** Mohamad Alameddine, Yara Mourad, Hani Dimassi

**Affiliations:** 1 Department of Health Management and Policy, Faculty of Health Sciences, American University of Beirut, Riad El-Solh, Beirut; 2 School of Pharmacy, Lebanese American University, Byblos, Lebanon; Örebro University, SWEDEN

## Abstract

**Background:**

Healthcare institutions have commonly reported exposure of employees, particularly nurses, to high levels of occupational violence. Despite such evidence in the Middle East Region, there is a dearth of national studies that have systematically investigated this phenomenon. This study investigates the prevalence, characteristics, consequences and factors associated with nurses’ exposure to occupational violence in Lebanon.

**Methods:**

A cross-sectional design was utilized to survey a nationally representative sample of 915 nurses registered with the Order of Nurses in Lebanon. Stratified random sampling by governorate was utilized. Individually-mailed questionnaires collected information on exposure to violence, degree of burnout and demographic/professional background. The main outcome variables were exposure to verbal abuse (never, 1–3, 4–9 and 10+ times) and physical violence (never, ever) over the past 12-months. Descriptive statistics were used to estimate prevalence of violence. Multivariable, binomial and multinomial regression models were carried out to investigate the correlates of exposure to verbal abuse and physical violence, respectively.

**Results:**

Response rate was 64.8%. Over the last year, prevalence of nurses’ exposure to verbal abuse was 62%, (CI: 58–65%) and physical violence was 10%, (CI: 8–13%). Among respondents, 31.7% of nurses indicated likelihood to quit their jobs and 22.3% were undetermined. Furthermore, 54.1% reported high levels of emotional exhaustion and 28.8% reported high levels of depersonalization. Compared to nurses with no exposure to verbal abuse, nurses reporting high exposure had high levels of emotional exhaustion (OR:6.4; CI:1.76–23.32), depersonalization (OR:6.8; CI: 3–15) and intention to quit job (OR:3.9; CI: 1.8–8.3). They further reported absence of anti-violence policies at their institutions (OR: 3; CI: 1.5–6.3). Nurses that were ever exposed to physical violence were more likely to be males (OR: 2.2; CI: 1.1–4.3), working day and night shifts (OR: 2.8; CI: 1.4–5.5) and subject to ten or more incidents of verbal abuse per year (OR: 46.7; CI: 10.1–214).

**Conclusions:**

An alarming two-thirds of respondents reported exposure to verbal abuse which was found to be a significant predictor of the three subscales of burnout, intention to quit and exposure to physical violence. The prevalence of exposure to physical violence is disconcerting due to its severe consequences. Policy and decision-makers are urged to use study findings for policy and practice interventions to create safe work environments conducive to nurses’ productivity and retention.

## Introduction

Occupational violence in healthcare settings is a global phenomenon [1,2]. It encompasses verbal abuse, physical assault and sexual harassment; with the former being the most commonly reported type of violence [3–5]. Nurses are consistently reported as being disproportionately exposed to violent incidents in the workplace [3,6–11].

Although relatives and patients are the most common perpetrators of violence [12,13], staff-to-staff violence (horizontal violence) is also reported in literature [14–16]. Determinants of violence classify under patients’ characteristics/profile, workplace and organizational characteristics and staff profile/healthcare workers variables [17,18]. Under patients’ characteristics and profile, mental illness, alcohol/ substance abuse and patient’s expectations are reported as predictors of violent behavior [7,19–21]. Waiting times, overcrowding and triage system have been reported among the most influential organizational characteristics linked to violent incidents [5,7,20]. The lack of clear anti-violence policies, poor security and uncontrolled movement of the public at hospitals were also suggested to contribute to patient’s violent attitudes and behaviors [5, 21,22]. Poor or inadequate allocation of staff, high attrition rates among experienced nurses, as well as high workload and lack of training are also influential organizational factors [5,22]. The type of the institution (as in private or public hospital) is also reported to contribute to violence with public hospitals reported to have a higher prevalence of violence compared to private ones [23]. With respect to staff profile, age, overall experience, gender and job tenure were identified as significant predictors of exposure to violence [19,24–27].

Consequences of workplace violence have been extensively investigated and are found to impact both the exposed nurses and the organization at large [10,28]. In addition to physical injury, nurses may be exposed to various emotional and psychological effects, including reduced self-esteem, fear, anxiety, depression, increased stress and burnout, mistrust, and difficulty with inter-personal relationships [10,15,28–30]. Exposure to violence may also affect the professional well-being of nurses, as manifested by increased absenteeism from work, impaired job performance, reduced job satisfaction and increased likelihood to leave the nursing profession [10,15,19,31,32]. Such consequences would also impact the organizational performance since staff well-being, productivity and quality of care are all inevitably affected [21,33,34]. All of these may culminate in sub-optimal outcomes of care, increased turnover rates and decreased profitability [28,35].

Despite the aforementioned critical consequences of exposure to violence, most staff members tend to underreport incidents [9]. This may be linked to a fear of retaliation, blame or stigma, as well as lack of confidence in the administration’s response [7,9,15,36]. More seriously, it could be related to nurses’ consideration of violence as an expected and tolerated aspect of their jobs [28,36,37], especially non-physical violence which is often perceived as a ‘norm’ in this profession [38] or as a “personal issue” [21].

Exposure to occupational violence in healthcare settings is a serious issue in the Middle East Region with several countries reporting prevalence of verbal and physical abuse that range from 48% to 86% and 7% to 42%, respectively [39–47]. In fact, a recent review revealed that the Middle East Region is reported to display the highest rate of nurse overall exposure to violence (61.3%), as compared to 38.3% in Europe during the same period [48].

The significance of this study is that it is the first national investigation of nurses’ exposure to occupational violence in Lebanon and a rare attempt to investigate such an exposure in the Middle East Region. The study builds on the findings of previous smaller scale institutional investigations which have confirmed that nurses are disproportionally exposed to occupational violence [47,49]. Furthermore, this study answers to recommendations of a national expert panel to implement a wider scale investigation across Lebanon [22]. The main objective of this study is to investigate the prevalence, characteristics and consequences of nurses’ exposure to occupational violence in Lebanese healthcare facilities.

## Methods

### Ethical approval

Ethical approval for this study and associated questionnaires and consent forms were sought from the Institutional Review Board (IRB) for Social and Behavioral Studies at the American University of Beirut. Protocol number FHS.MA.12. All participating nurses signed a written consent form that was approved and stamped by the IRB office.

### Study Design

A cross-sectional design was utilized to survey a stratified random sample of nurses registered with the Order of Nurses in Lebanon. Each stratum represented one of the six main administrative divisions/regions of the country called governorate.

The sampling frame for this study was individual nurses and the main outcome variables were exposure to verbal abuse and/or physical violence. Nurses were eligible to be included in the sampling frame for this study if they were registered with the Order of Nurses as of December 31, 2011 –the sampling frame was acquired from the Order of Nurses. Nurses should also have a valid geographic address in their profile so that they could be allocated to a particular stratum.

The original database included the records of 7799 nurses. We excluded from the sampling frame 1220 nurses because they did not specify their place of residence, governorate or district. The final database included the records of 6579 nurses. In order to estimate the number of subjects needed for this study, the estimated true prevalence, the desired level of precision (acceptable error) and the level of confidence were evaluated. Sample size determination was based on the single proportion estimation formula with 95% confidence interval and 4% estimation precision. The research team decided to select the most conservative estimate of prevalence of violence (50%), whether verbal or physical, for sample size calculation known to produce the largest sample size. A minimum of 558 respondents were necessary. To account for wrong addresses and non-respondent the authors elected to draw a random sample of 915 nurses from various governorates.

Using the Order of Nurses registration database, the proportional of nurses for each Governorate was calculated. Then the research team used the probability proportional to size in identifying the needed number of nurses from each of the six Lebanese governorates. Using a separate datasheet listing all registered nurses for each of the governorates, the required number of nurses was randomly selected using a sampling software. The records of all sampled nurses were validated and contact information checked and updated.

### Survey instrument

The survey instrument included a structured questionnaire collecting data on four main sections: (1) demographic and professional background, (2) exposure to and consequences of violence-Verbal Abuse and Physical Violence over the last 12 months (3) intention to quit and (4) level of burnout using the Maslach Burnout Inventory (MBI) Human Services Survey with a twelve months recall period [50]. Note that for the purpose of this study verbal abuse refers to threats of violence without actual physical contact, threatening or harassing behaviors, emotional abuse and emotional aggression. Physical violence, on the other hand, refers to physical assault, beatings, punching, kicking, biting, spitting, or any form of physical aggression [6].

Exposure to verbal abuse was reported on a four-point frequency scale (never, 1–3 times, 4–9 times and 10+ times within the past twelve months), while exposure to physical violence was reported as ‘never’ or ‘ever’ within the past twelve months.

The original version of the questionnaire was prepared in English. To enhance the content validity of the survey instrument, it was reviewed and amended by a panel of experienced professionals chosen due to familiarity of the topic, including a representative from the Order of Nurses, a nursing researcher, an occupational health researcher, a statistician and an experienced nurse. The final version of the questionnaire was translated from English to Arabic by an experienced translator and then back-translated by another translator to English. No significant variations were identified. Both language versions of the questionnaire were pilot-tested on 50 nurses each, who were excluded from the study.

### Data collection

Nurses were surveyed in the spring and summer of 2012. Nurses were first contacted by phone to be informed about the study and invited to participate. A total of 81 nurses refused to participate due to their busy schedule; numbers were distributed across the regions. Nurses agreeing to participate were then mailed out the survey questionnaire to an address of their choice. Nurses were instructed to complete the questionnaire in person at a place of privacy and place them in sealed envelopes that were provided by and addressed to the research team. Completed questionnaires were picked up by a secure courier. No personal identifiers were used. A total of 593 nurses returned the questionnaires (response rate of 64.8%).

### Data analysis

After coding, the data was analyzed using the Statistical Package for Social Science (SPSS), version 19.0. First level analysis consisted of summarizing sample characteristics, exposure to and characteristics of violence, intention to quit and MBI burnout subscales. The second level of analysis looked at the factor associations with exposure to violence, verbal and physical separately, using the Pearson chi-square. The third level of analysis consisted of the multivariate model whereby exposure to verbal and physical violence were the dependent variables (each in a separate model), and factors identified to be statistically significant in the bivariate analysis were included to control for their potential confounding effect. Only independent variables that remained statistically significant were kept in the final models.

Since exposure to verbal abuse was reported on a four-point frequency scale a multinomial logistic regression modeling technique was utilized. Exposure to physical violence, on the other hand, was reported as ‘never’ or ‘ever’ and was modeled using the binary logistic regression techniques. Coefficients and standard errors produced were exponentiated to create OR and their respective 95% confidence intervals.

## Results

### Sample description

As shown in Table 1, the majority of the nurse respondents in the study were females (79.4%), aged between 25–34 years (51.6%) and married (53.6%). A total of 59.2% of respondents held a university degree and an evident majority (91.4%) had a permanent full-time employment, with most nurses (53.1%) working day shifts. Note that differences in nurses’ exposure to violence by geographic region were not observed in this study.

**Table 1:** Demographic and professional characteristics of survey respondents (n = 593).

Variable	Options	N (%)
**Gender**	**Male**	122 (20.6%)
	**Female**	471 (79.4%)
**Age Group**	**<25 years**	95 (16.0%)
	**25–34 years**	306 (51.6%)
	**35–44 years**	111 (18.7%)
	**45–54 years**	65 (11.0%)
	**Missing**	16 (2.7%)
**Marital Status**	**Single**	259 (43.7%)
	**Married**	318 (53.6%)
	**Others**	11 (1.9%)
	**Missing**	5 (0.8%)
**Degree Type**	**University**	351 (59.2%)
	**Non-university/Technical**	237 (40.0%)
	**Missing**	5 (0.8%)
**Working Status**	**Permanent- Full time**	542 (91.4%)
	**Permanent- Part time**	18 (3.0%)
	**Temporary/Casual**	24 (4.0%)
	**Missing**	9 (1.5%)
**Type of Shifts**	**Mostly Day Shifts**	315 (53.1%)
	**Mostly Night Shifts**	77 (13.0%)
	**Day & Night Shifts-Rotation**	194 (32.7%)
	**Missing**	7 (1.2%)

### Exposure to verbal abuse

Table 2 provides a summary of respondents’ reported exposure to and characteristics of verbal abuse over the past twelve months. The table shows that 62% (CI: 58–65%) of nurses reported exposure to at least one incident of verbal abuse, with 80% of incidences occurring during day shifts. With regards to the site of the last incident of verbal abuse, 34.7% took place inside patients’ rooms, while 22.7% and 11.4% occurred in hallways and at the nursing station, respectively. The most common types of reported verbal abuse were ‘being shouted at’ and encountering episodes of ‘angry outbursts’. Several instigators of verbal abuse were identified by respondents, with the most frequently expressed factor being staff attitude (44.4%). Instigators related to patients and patients’ families included traumatized family members and friends (35.6%) and unrealistic expectations of patients (35.3%). Most common perpetrators of verbal abuse were patients’ family/friends (39.4%), patients (25.2%), medical staff (24.6%), nursing staff (18.1%) and manager/supervisor (11.3%).

**Table 2:** Nurses' exposure to and characteristics of verbal abuse and physical violence.

Variable	Verbal Abuse (total 587)		Physical Violence (total 572)	
	Values	%	Values	%
**Exposure to verbal abuse in past 12 months**	Never	38.2	Never	90.4
	1–3 times	37.3	Ever	9.6
	4–9 times	13.5		
	10 +	11.1		
**Timing of the last incident of verbal abuse**	Day shift	80.4	Day shift	50
	Night shift	19.6	Night shift	50
**Location of the last incident of verbal abuse**	Patient room	34.7	Patient room	54.7
	Hallway	22.7	Hallway	17
	Nursing station	11.4	Examination room	7.5
	Waiting room	6.5	Triage room	3.8
	Examination room	6.3	Waiting room	3.8
	Other places	19.3	Other places	13.2
**Most common types of verbal abuse** *	Loud noises and shouting	73.9	Pushing	50.9
	Angry outbursts	48.7	Grabbing/punching	37.7
	Demanding/threatening/sarcastic/condescending comments	32.9	Kicking	22.6
			Attack with any tool	15.1
			Spitting	11.3
	Swearing and cursing	25.8	Biting	5.7
	Threats	8.5	Attack with weapon	1.9
**Top instigators of verbal abuse** *	Staff attitude	44.4	Unrealistic expectations by patients	41.5
	Traumatized family members/friends	35.6	Traumatized family members/friends	28.3
	Unrealistic expectations by patients	35.6	Mental health condition	26.4
	Mental health condition	9.3	Alcohol/drug abuse	7.5
	Alcohol/drug abuse	3.4	Staff attitude	5.7
**Top perpetrators of verbal abuse** *	Patient family/friend	39.4	Patient	53.6
	Patient	25.2	Patient family/friend	33.9
	Medical staff	24.6	Public	8.9
	Nursing staff	18.1	Manager/supervisor	3.6
	Manager/supervisor	11.3	Medical staff	1.8
	Public	2.3	Nursing staff	1.8

*Note that categories in this question are not mutually exclusive

### Exposure to physical violence

In terms of exposure to physical violence, Table 2 reveals that 10% (CI: 8–13%) of surveyed nurses reported being subjected to physical violence. Exposure was equally distributed between the day and night shifts and the most reported incidents took place in the patients’ rooms (54.7%). The most common types of assaults recounted were pushing, grabbing/punching, and kicking. Attack with a tool and attack with a weapon were cited types of physical violence by 15.1% and 1.9% of respondents, respectively. The top reported instigators of physical violence were unmet expectations of patients (41.5%) and traumatized family members/friends (28.3%), and perpetrators were mostly patients or patient family and/or friends.

### Outcomes associated with and available policies on violence

Respondents reported various encountered outcomes that resulted from their exposure to violence, mainly difficulty with family relationships (32.3%), difficulty sleeping (31.3%) and appetite problems (10.5%). Their top reported responses to exposure included reporting the incident to the supervisor (56.0%), exercising self-defense (49.4%), considering leaving the institution (21.3%), and reporting the incident to family members, friends or colleagues (21.3%). Only 17.7% of respondents indicated completing an incident report, and only 1.8% reported the incident to the police. Only 46% of nurses indicated the presence of antiviolence regulations and policies at their workplace.

### Burnout and intention to quit

While less than half (46.0%) indicated that is it unlikely for them to quit their current employment within the next 12 months, Close to one third of respondents (31.7%) expressed intention to quit (Table 3). Table 3 also reveals that reported high level of professional burnout was related to emotional exhaustion (EE), depersonalization (DP) and personal accomplishment (PA) in 54.1%, 28.8% and 24.1% of respondents, respectively.

**Table 3:** Intention to quit and level of burnout among surveyed nurses.

Variable	Values	Percentage (%)
**Intention to quit your job over next 12 months**	Likely	**31.7**
	Don’t know/not sure	22.2
	Unlikely	46
**Burnout subscale: Emotional exhaustion (EE)**	**High**	**54.1**
	Medium	20.6
	Low	25.3
**Burnout subscale: Depersonalization (DP)**	**High**	**28.8**
	Medium	20.2
	Low	51
**Burnout subscale: Personal accomplishment (PA)**	**High**	**24.1**
	Medium	26.5
	Low	49.3

### Bivariate analyses on exposure to verbal abuse

As originally hypothesized, exposure to verbal abuse was significantly associated with the three subscales of burnout—EE (p-value<0.001), DP (p-value<0.001), and PA (p-value = 0.014), where nurses that had moderate and high levels of burnout on the sub-scales were more likely to have been exposed to at least one episode of verbal abuse. Moreover, a significant association was observed between exposure to verbal abuse and intention to quit (p-value<0.001), whereby those who reported intention to quit were more likely to have been subjected to verbal abuse; this was consistent across all frequencies of exposure.

With regards to age, analysis reveals that the relatively younger nurses (<34 years old) were more likely to have been exposed to verbal abuse. Single nurses were also more likely to have been exposed to verbal abuse compared to married nurses (p-value = 0.017). The years of experience variable was also significantly associated with verbal abuse (p-value = 0.004), with the least experienced nurses (less than 5 years of experience) reporting the highest rate of exposure to 4–9 episodes over the last 12 months. Furthermore, nurses working rotations of day and night shifts were more likely to report exposure to verbal abuse as compared to those working only day or night shifts (p-value = 0.039). Moreover, exposure to verbal abuse was significantly linked to the presence of specific anti-violence policies and regulations at the organization (p-value<0.001).

### Bivariate analyses on exposure to physical violence

Analysis reveals that nurses who have been subjected to physical violence were more likely to have moderate or high burnout related to depersonalization (p-value = 0.026). Nurses who reported ever being subjected to physical violence were more likely to be males (p-value = 0.001) and aged less than 34 years (p-value = 0.046). In terms of length of service, the highest proportion of nurses exposed to physical violence had worked for up to 9 years, after which occurrence of violence declined (p-value = 0.042). Those exposed were more likely to work rotations of day and night shifts; moreover, when comparing day shifts to night shifts, incidents of physical violence were more likely to occur during night shifts (p-value<0.001).

Last but not least, analysis established a significant association between exposures to physical violence and verbal abuse (p-value<0.001). Nurses reporting exposure to physical violence were more likely to have been verbally abused.

### Multivariate analyses

A multinomial logistic regression model with exposure to verbal abuse as the dependent variable is reported in Table 4. Analysis reveals that the EE and DP subscales of burnout were significantly associated with exposure to verbal abuse. Nurses with high level of burnout on the EE subscale had 6.41 times the odds of being verbally abused more than ten times during the past twelve months as compared to respondents with low level of EE (95% CI = 1.76–23.32; p-value 0.005). Compared to those with low level of DP, respondents with a high level of burnout on the DP subscale had 1.90 times the odds of being verbally abused one to three times (95% CI 1.09–3.27; p-value 0.023), 3.83 times the odds of being verbally abused four to nine times (95% CI 1.88–7.80; p-value <0.001) and 6.75 times the odds of being verbally abused more than ten times (95% CI 3.04–15.00; p-value <0.001). Analysis also reveals that when compared to those who indicated unlikelihood to quit, respondents who expressed likelihood to quit their jobs had 2.64 times the odds of being verbally abused one to three times (95% CI 1.58–4.43; p-value <0.001), 2.33 times the odds of being verbally abused four to nine times (95% CI 1.15–4.72; p-value 0.020) and 3.85 times the odds of being verbally abused more than ten times (95% CI 1.78–8.32; p-value 0.001).

**Table 4:** Multinomial logistic regression model on exposure to verbal abuse.

	Verbal Abuse					
	1–3 times vs. Never		4–9 times vs. Never		10+ times vs. Never	
	OR (95% CI)	P-value	OR (95% CI)	P-value	OR (95% CI)	P-value
**Emotional Exhaustion (High)**	1.35 (0.78–2.34)	0.289	2.27 (0.92–5.61)	0.075	6.41 (1.76–23.32)	**0.005**
**Emotional Exhaustion (Medium)**	1.40 (0.77–2.53)	0.262	1.78 (0.66–4.83)	0.258	1.80 (0.39–8.21)	0.449
**Depersonalization (High)**	1.90 (1.09–3.27)	**0.023**	3.83 (1.88–7.80)	**<0.001**	6.75 (3.04–15.00)	**<0.001**
**Depersonalization (Medium)**	1.84 (1.07–3.17)	**0.028**	2.14 (0.97–4.70)	0.060	3.09 (1.23–7.73)	**0.016**
**Intention to Quit (Likely)**	2.64 (1.58–4.43)	**<0.001**	2.33 (1.15–4.72)	**0.020**	3.85 (1.78–8.32))	**0.001**
**Intention to Quit (Not sure)**	1.28 (0.75–2.16)	0.366	1.23 (0.57–2.61)	0.600	1.25 (0.52–3.00)	0.613
**Absence of anti-violence policies and regulations**	1.25 (0.82–1.89)	0.303	1.05 (0.58–1.89)	0.871	3.13 (1.54–6.25)	**0.002**

Additionally, analysis reveals that nurses reporting absence of anti-violence policies had 3.13 times the odds of being exposed to a high frequency of verbal abuse (95% CI 1.54–6.25; p-value 0.002) when compared to those who confirmed the presence of such policies.

Table 5 displays the results of a binary logistic regression examining the factors significantly associated with physical violence. Analysis reveals that male nurses had 2.22 times the odds of exposure to physical violence compared to their female counterparts (95% CI 1.14–4.35; p-value 0.019). Nurses who reported working day and night shifts had 2.78 times the odds of exposure to physical violence when compared to those working day shifts only (95% CI 1.40–5.54; p-value 0.004). Those exposed to verbal abuse had higher odds of exposure to physical violence compared to those with no exposure to verbal abuse, with the highest odds (46.68 times) associated with the highest frequency of exposure to verbal abuse (95% CI 10.18–214.0.7, p-value <0.001).

**Table 5:** Binary logistic regression model on exposure to physical violence.

Variable	Physical Violence	
	Ever vs. Never	
	OR (95% CI)	P-value
**Gender (Male)**	2.22 (1.14–4.35)	**0.019**
**Work Shift (Night)**	1.57 (0.60–4.16)	0.363
**Work Shift (Day-Night Rotation)**	2.78 (1.40–5.54)	**0.004**
**Verbal Abuse (1–3 times)**	10.65 (2.43–46.76)	**0.002**
**Verbal Abuse (4–9 times)**	29.73 (6.47–136.59)	**<0.001**
**Verbal Abuse (10+ times)**	46.68 (10.18–214.07)	**<0.001**

## Discussion

This paper presents the first national attempt to systematically investigate exposure of Lebanese nurses to occupational violence and the factors associated with such exposure. Findings confirm a high level of nurses’ exposure to occupational violence, with two out of each three nurses reporting exposure to verbal abuse and one of each ten reporting exposure to physical violence, over the past twelve months. Study findings are in conformity with other studies carried out in the region [29,39]

The reported frequency of exposure to physical violence is highly worrisome when such events involve the presence of a weapon, as was reported by 2% nurses exposed to physical violence. Additionally, under the assumption that our study sample is representative of all nurses in Lebanon, the current prevalence of exposure to physical violence observed amongst respondents would be translated into 750 incidents of physical violence over a one year period.

Exposure to violence, whether in the form of verbal abuse or physical violence, resulted in detrimental consequences to exposed nurses in this study. Moreover, the recounted outcomes may have been the underlying causes behind the observed high level of burnout related to all three subscales (EE, DP, and PA), in addition to the alarming finding of a 32% intention to quit. These findings are consistent with those of other studies on workplace violence, in which exposure to violent incidents, both verbal and physical, has resulted in increased burnout often associated with higher turnover rates [35,51,52]. Such issues, if not tackled promptly, may aggravate the reported nursing shortages in Lebanon [53,54].

Furthermore, the study findings reveal that exposure to verbal abuse was a significant predictor of exposure to physical violence, professional burnout, as well as a higher intention to quit. As the frequency of exposure to verbal abuse increased, the odds of exposure to physical violence increased drastically, a finding that is concurrent with other studies [29,45,55,56]. Moreover, the significant association between exposure to verbal abuse and all three subscales of burnout is quite disconcerting. Furthermore, nurses who expressed an intention to quit were significantly more likely to have been subjected to verbal abuse, across all levels of frequency of exposure. Attention must therefore be exerted towards protecting the nursing profession from the adverse impact of exposure to verbal abuse which should no longer be regarded as a tolerated aspect of the work environment.

Younger nurses in this study (aged 34 years or less) were more likely to have been subjected to either forms of violence; a finding consistent with other studies [55,57–59]. This association was further supported by the finding related to length of service, where nurses with shorter years of experience (less than nine years) were more likely to be exposed [60]. This could be attributed to younger nurses’ inexperience in managing violent situations [33,61]. The finding may also be attributable to the “healthy worker effect”, where nurses who did not experience violence or are better able to deflect violent incidents may have a higher propensity in remaining in the active workforce.

Analysis further revealed that verbal abuse was occurring horizontally while physical violence was more vertical in nature. Verbal abuse was most commonly instigated by attitude of co-workers and enacted by staff members. Physical violence, however, mostly took place in the patients’ rooms and by patients or patients’ family members and/or friends, such findings concur with the findings of other studies [15,29,36,37,55,62–64].

Adverse consequences of horizontal or lateral violence have been well-documented and extend to turnover [15,16,65,66]. Furthermore, hostility from coworkers has been shown to be more frustrating to cope with than patient violence and is associated with reduced self-esteem [67,68]. It is, therefore, the duty of institutional leaders and managers to investigate and address the root causes behind horizontal violence and foster a culture of reporting violent incidents, especially if they come from a co-worker.

### Limitations

The study has a number of limitations that warrant mentioning. First, the 12-months recall period may have increased the risk of recall bias and resulted in nurses’ either over or under reporting of incidents of exposure to violence. Second, the cross-sectional nature of the study does not support the establishment of causal relationships but rather the presence of significant associations with the outcome variables, establishment of causality will require studies that are longitudinal in nature. Third, although the study had a satisfactory response rate, it could not be ascertained whether non-responding nurses had a different experience with violence compared to respondents.

### Policy and practice implications

Stakeholders including the Ministry of Public Health, Orders, Syndicates and policy makers are further urged to consider these findings as they reflect a dangerous work culture that could jeopardize the safety of staff members and may have negative consequences on patients’ care and outcomes.

At the policy level, the study unearthed the absence of antiviolence policies and regulations in the healthcare facilities of most responding nurses. Such a finding added to the observed significant association between exposure to violence and absence of antiviolence policies may increase the risk of nurses’ exposure to occupational risks. Study findings strongly support the need to implement the recommendation of the International Council of Nurses to incorporate a special anti-violence section in the occupational health chapter of the national accreditation requirement of Lebanese healthcare facilities [69].

At the institutional level, leaders and managers should endorse, implement and evaluate an institutional zero tolerance policy for violence. Such a policy has already been mandated by various national and international agencies for workplace safety and has been found to decrease violence in the workplace [1,70]. The successful implementation of antiviolence policies necessitates their incorporation into comprehensive violence prevention programs that include well-established reporting systems and post-incident support for subjected personnel [71]. In addition, they should support, in collaboration with professional orders and syndicates, staff training programs on how to prevent and manage exposure to occupational violence in health care institutions, as well as team building workshops in order to mitigate the effects of horizontal violence. Such programs have shown satisfactory results, in terms of enhanced knowledge, skills and attitudes in successfully handling aggressive patients [72].

## Conclusion

Exposure of nurses to occupational violence is a global phenomenon; our study revealed that Lebanon is no exception in that regard. Yet, the study identified multiple opportunities for intervention at the policy and practice level and highlighted an urgent agenda for action by leaders, managers and concerned stakeholders. Although some of the identified association and trends and associated recommendations might be context specific, many will be applicable to other countries in the region. It is acknowledged that change will not happen overnight and that many of the recommendations will take time to be implemented, yet the findings of this study are clear; there is a wide margin for intervention in order to protect nurses from occupational violence at multiple levels.
